# Breast-lesion assessment using amide proton transfer-weighted imaging and dynamic contrast-enhanced MR imaging

**DOI:** 10.2478/raon-2023-0051

**Published:** 2023-11-30

**Authors:** Lulu Zhuang, Chun Lian, Zehao Wang, Ximin Zhang, Zhigang Wu, Rong Huang

**Affiliations:** Department of Radiology, Peking University Shenzhen Hospital, Shenzhen, China; Shantou University, Shantou University Medical College, Shantou, China; Clinical & Technical Support, Philips Healthcare (Shenzhen) Ltd., China

**Keywords:** amide proton transfer, breast lesions, dynamic contrast-enhanced, magnetic resonance imaging

## Abstract

**Background:**

Previous studies have indicated that amide proton transfer-weighted imaging (APTWI) could be utilized for differentiating benign and malignant tumors. The APTWI technology has increasingly being applied to breast tumor research in recent years. However, according to the latest literature retrieval, no relevant previous studies compared the value of APTWI and dynamic contrast-enhanced (DCE) magnetic resonance imaging (MRI) in distinguishing benign lesions from malignant lesions. In the present study, the application of APTWI and DCE for differentiating the benign and malignant breast lesions was investigated.

**Patients and methods:**

APTWI was performed on 40 patients (42 lesions) who were enrolled in this prospective study. The lesions were split into two groups, one with malignant breast lesions (n = 28) and the other with benign breast lesions (n = 14), based on the results of the histology. The measured image characteristics (APT value, apparent diffusion coefficient [ADC] value, and time-of-intensity-curve [TIC] type) were compared between the two groups, and the ROC curve was used to quantify the diagnostic performance on the basis of these factors. The correlation between the APT values and the estrogen receptor (ER), progesterone receptor (PR), human epidermal growth factor receptor 2 (HER-2), and Ki-67 expression levels and histological grades was examined using Spearman's correlation coefficient.

**Results:**

The measured APT and ADC values showed a strong inter-observer agreement according to the intraclass correlation coefficients (0.954 and 0.825). Compared to benign lesions, malignant lesions had significantly higher APT values (3.18 ± 1.07 and 2.01 ± 0.51, p < 0.001). Based on APTWI, DCE, diffusion-weighted imaging (DWI), and ADC + APTWI, ADC + DCE, and DCE + APTWI, the area-under-the-curve values were 0.915, 0.815, 0.878, 0.921, 0.916, and 0.936, respectively.

**Conclusions:**

APTWI is a potentially promising method in differentiating benign and malignant breast lesions, and may it become a great substitute for DCE examination in the future.

## Introduction

Breast cancer is the most prevalent malignant neoplasm all over the world.^1^ Magnetic resonance imaging (MRI), a non-invasive technique with exceptional soft tissue resolution, plays a significant role in diagnosis, treatment, and prognosis assessment of breast diseases.^2,3,4^ In an effort to standardize the imaging strategy for breast lesions, the American College of Radiology created the Breast Imaging Reporting Data System (BI-RADS) in 1992.^5^ However, some imaging features of benign and malignant lesions still overlap. False-positive results from conventional breast MRI could result in unnecessary invasive biopsies being performed.^6^

Dynamic contrast-enhanced MRI (DCE-MRI) is used to obtain focal information by injecting a contrast agent, and it has been widely used for determining prognosis, monitoring therapy, and diagnosing many diseases.^7^ The study of Alkhunizi SM *et al*. provided insights into the consequences of gadolinium-based contrast agent (GBCA) delivery by showing considerable retention of gadolinium in the spinal cord and peripheral nerves 1 day after dose.^8^ Meanwhile, after multiple GBCA injection, the dentate nucleus and pallidum showed abnormally high signals under T1-weighted imaging sequence.^9^ Therefore, the search for a safe tool for evaluating breast lesions is of great importance.

In 2000, the first MR contrast images of several small molecules were acquired by Wolff *et al*., who called this novel molecular imaging technology chemical exchange saturation transfer (CEST).^10^ Amide proton transfer-weighted imaging (APTWI) technique is a new molecular MRI sequence that is based on CEST, focusing on the exchange between amide protons and bulk water and thus generating image contrast at 3.5 parts per million (ppm) away from water frequency.^11^ Various preclinical and clinical research investigations have been conducted on this imaging technology^12^, making it a potential molecular imaging tool that is now used in clinics.^13^ Meanwhile, in recent years, the differentiation of tumor subtypes and grades and the assessment of therapy efficacy are two applications of APTWI in breast cancer that have drawn increasing attention.^14,15,16,17^ However, no research has compared the value of APTWI in diagnosing benign and malignant breast diseases with it in DCE.

The purpose of this study was to investigate the potential of APTWI in breast-lesion diagnosis and compare the performance of DCE and APTWI in identifying benign from malignant breast lesions.

## Patients and methods

### Patients

The local Ethics Committee of Peking University Shenzhen Hospital granted approval for this prospective trial (reference number 2022-073) and each subject provided their informed consent.

**FIGURE 1.**
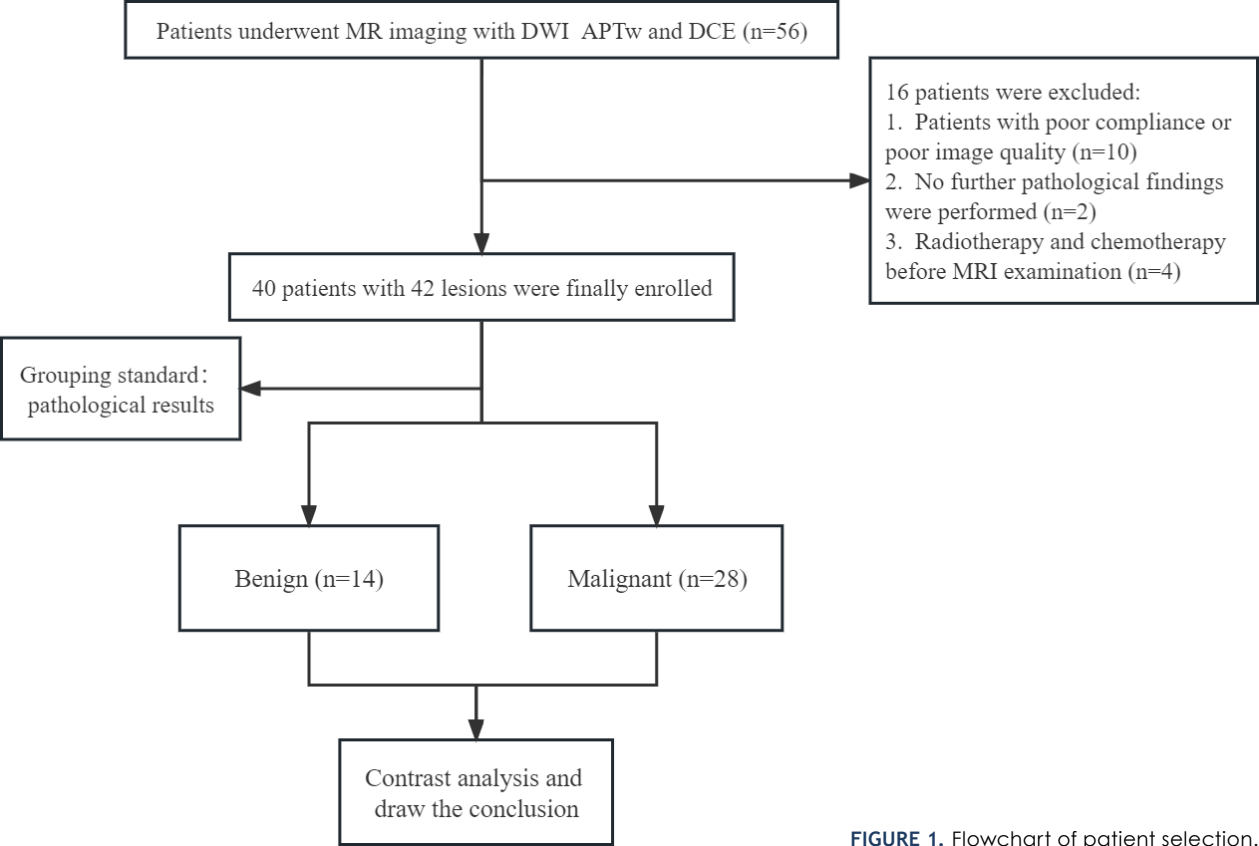
Flowchart of patient selection.

Patients with mammary lesions were enrolled from June 2022 to December 2022 in compliance with the following standards: 1) no contraindication to MRI examinations; 2) suspicious breast lesions discovered by mammogram and/or ultrasound; 3) no previous surgical procedure, chemotherapy, or radiotherapy before MRI examination; 4) the histopathology of each mammary lesion was validated by a biopsy or surgical specimen. The flowchart of patient enrollment is represented in Figure 1.

### MR examination

The entire examinations were conducted on a 3.0T MRI scanner (Ingenia CX, Philips Healthcare) equipped with a 16-channel breast coil. Both breasts were draped naturally over the center of the coil while the patients were lying in the prone position with the feet entering first. The traditional sequences, widely used in clinical practice, were performed firstly, including T1-weighted imaging, T2-weighted imaging, and diffusion-weighted imaging (DWI). Following completion of the traditional sequences, all slices containing lesion tissues were subjected to three-dimensional (3D) APTWI examinations using the images from the traditional sequence as the reference. Finally, DCE was performed with the injection of GBCA (gadodiamide; 0.2 mL/kg body weight; Bayer AG). Table 1 lists the imaging parameters in detail.

**TABLE 1.** Imaging protocol parameters

	**APTw**	**DWI**	**DCE**
**TR [ms]**	5500	6356	4
**TE [ms]**	9	84	2
**Field of views [mm^2^]**	120 × 120	340 × 309	260 × 340
**Voxel size**	1.9 × 2.0 × 7	2.7 × 2.3 × 4	0.9 × 0.9 × 3.5
**Flip angle**	90	90	11
**Matrix (mm^2^)**	64 × 60	128 × 133	280× 368
**Reconstructed voxel size**	0.94 × 0.94 × 7	0.97 × 0.97 × 4	0.61 × 0.61 × 3.5
**b-value [s/mm^2^]**	NA	0,50,800	NA
**Bandwidth [pixel/Hz]**	406.9	23.1	783.2
**Scan duration**	5 min 03 s	2 min 14 s	7 min 44 s

APTWI = amide proton transfer-weighted imaging; DCE = dynamic contrast enhancement; DWI = diffusion-weighted imaging; NA = not applicable

### Imaging analysis

All data were analyzed using the IntelliSpace Portal (Philips Healthcare, Cleveland, OH, USA) workstation. First, the APTWI pseudo-color images were merged with the DCE images. Next, using the plain scan and DWI used as references, regions of interest (ROIs) were manually delineated by highlighting the solid portion of the lesion tissue on the axial DCE images. On the DCE images, on the slice that showed the largest lesion area, the capsule's necrotic and hemorrhagic areas were kept as far away from as possible.^18^ Two radiologists separately assessed the ROI (L.Z. and R.H., with 3 and 25 years of breast imaging diagnostic experiences, respectively). For the purpose of measuring apparent diffusion coefficient (ADC) value, the identical ROI was converted to an ADC image. The formula below was used to calculate the APT value:

APT value = MTRasym (3.5 ppm) (%) = [Ssat (−3.5 ppm) − Ssat (+3.5 ppm)]/S0

Note: Ssat = the signal intensity after applying the saturation pulse; S0 = the signal intensity without the saturation pulse; MTRasym (3.5 ppm) = the magnetization transfer ratio asymmetry at 3.5 ppm

Time–intensity curves (TICs) were generated from the DCE images, and they were separated into three categories: I = Persistent, II = Plateau, and III = Washout. Disagreements regarding the type of TIC were resolved by discussion. On the DCE images, the maximum diameter of each lesion was measured.

### Pathological grade and stage

All diagnoses of breast lesions were confirmed by biopsy or surgical histopathology. According to pathological standards, grade I breast cancer was defined as well-differentiated tumors. Grades II and III refer to moderately and poorly differentiated tumors, respectively. The following were the explanation criteria for the estrogen receptor (ER) and progesterone receptor (PR) status: ≥ 10 percent of the tumor cells exhibited positive results, while < 10 percent showed negative results.^19^ The human epidermal growth factor receptor-2 (HER-2) expression status was considered as positive when samples scored +++ or when HER2 gene amplification was proven in case of a ++ score.^20^ A critical point of 14% separates high-expression and low-expression values for Ki-67.^21^

### Statistical analysis

SPSS 19.0 (IBM) and MedCalc 20.0 were used to analyze the data. In order to evaluate inter-observer reliability, the intra-class correlation coefficient (ICC) was used. Kolmogorov–Smirnov test was utilized to ascertain if the quantitative data followed a normal distribution. The variations in each parameter across several groups were compared using t-test or Mann–Whitney U test. Each parameter's diagnostic efficacy was assessed using ROC curves. Meanwhile, Delong tests were used to evaluate whether one parameter's area under the ROC curve (AUC) differed from the others. By using Spearman's correlation coefficient, the correlations between APT values and various clinicopathological variables were calculated. The composite diagnosis of multiple indices was determined using a logistic regression. P < 0.05 was considered statistically significant.

## Results

### Clinicopathological data

According to the inclusion and exclusion criteria, a total of 40 patients with 42 lesions were enrolled. All lesions were classified into two groups: benign group (n = 14; including seven fibroadenomas, one juvenile fibroadenoma, two adenoses, one intraductal papilloma, one benign phyllodes tumor, and two fibroadenomas mixing adenoses) and malignant group (n = 28; including 24 invasive breast carcinomas and four ductal carcinoma in situ). The summary of patient characteristics and the clinical and pathological data of breast cancer are displayed in Tables 2 and 3, respectively.

**TABLE 2.** Summary of patient characteristics

	**Benign lesions (n = 14)**	**Malignant lesions (n = 28)**
**Age (years)**	35 ± 14 (13–68)	49 ± 12 (30–77)
**Largest diameter (cm)**	2.19 ± 2.40	3.31 ± 1.25
**Histology**	Fibroadenoma (n = 7)	Invasive breast carcinoma (n = 24)
	Juvenile fibroadenoma (n = 1)	Ductal carcinoma in situ (n = 4)
	Adenosis (n = 2)	
	Fibroadenoma + adenosis (n = 2)	
	Intraductal papilloma (n = 1)	
	Benign phyllodes tumor (n = 1)	

**TABLE 3.** Clinical and pathological data of breast cancer

**Variable**	**Malignant breast tumors (n = 28)**
**Tumor diameter**	
< 2 cm	3 (10.71)
≥ 2 cm	25 (89.29)
**Metastatic status of axillary lymph node**	15 (53.57)
**Calcification**	16 (57.14)
**Grade of IBC (N, %)**	
Grade I	1 (4.17)
Grade II	12 (50)
Grade III	11 (45.83)
**Receptor status (N, %)**	
ER+	20 (71.43)
PR+	20 (71.43)
HER-2+	9 (32.14)
Ki67+	25 (89.29)

One patient without the result of immunohistochemical

ER = estrogen receptor; HER-2 = human epidermal growth factor receptor-2; PR = progesterone receptor

### Consistency test

The two observers had solid agreements. The ICCs were 0.954 for the APT values and 0.825 for the ADC values. As a result, the final evaluation indices were calculated using the averages of the two radiologists’ measurements of the parameters.

### Comparison of MRI parameters

**FIGURE 2.**
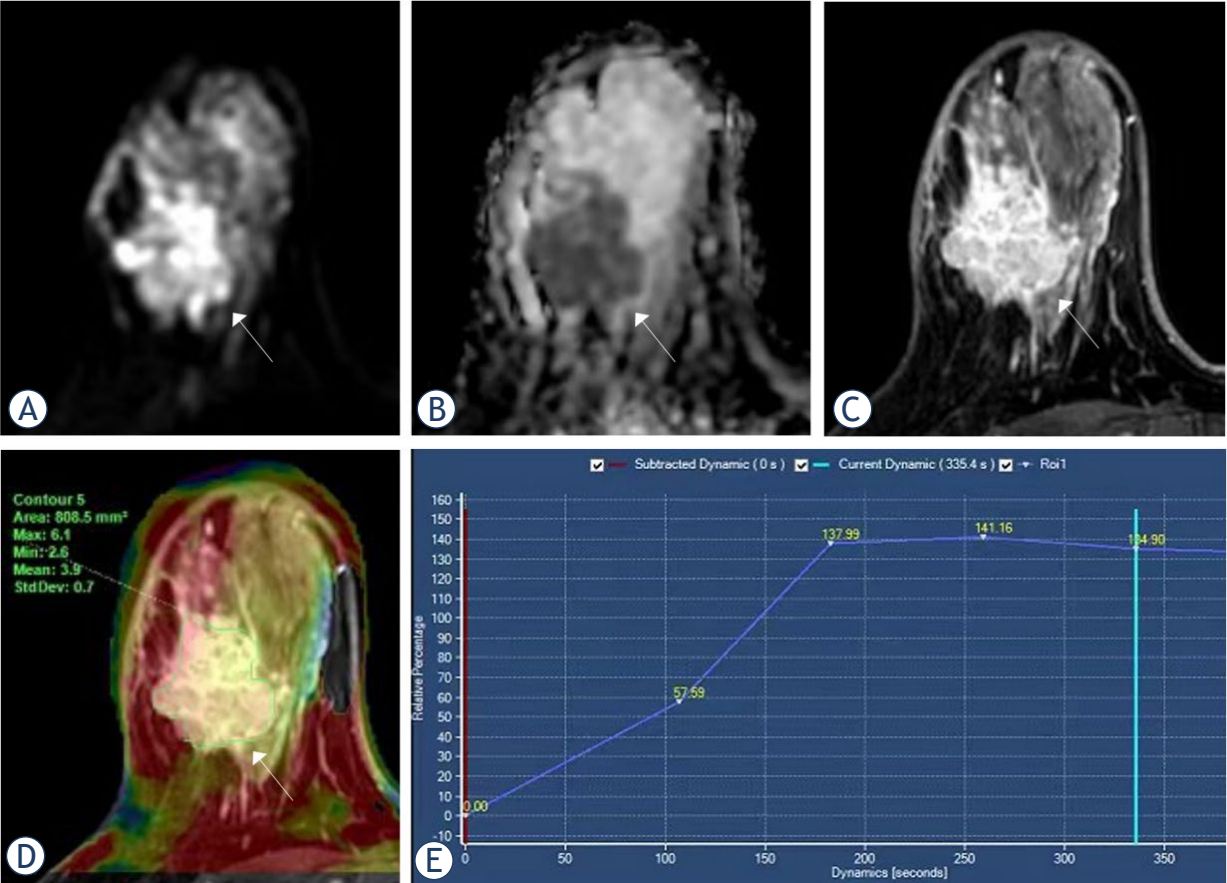
Magnetic resonance imaging (MRI) from a 42-year-old woman with invasive breast carcinoma. **(A)** DWI = diffusion-weighted imaging **(B)** apparent diffusion coefficient (ADC) **(C)** dynamic contrast-enhanced (DCE) **(D)** The amide proton transfer-weighted imaging (APTWI) pseudo-color image was merged with the DCE images and the APT value was 3.9%. **(E)** The type of time-intensity curves (TIC) was plateau.

**FIGURE 3.**
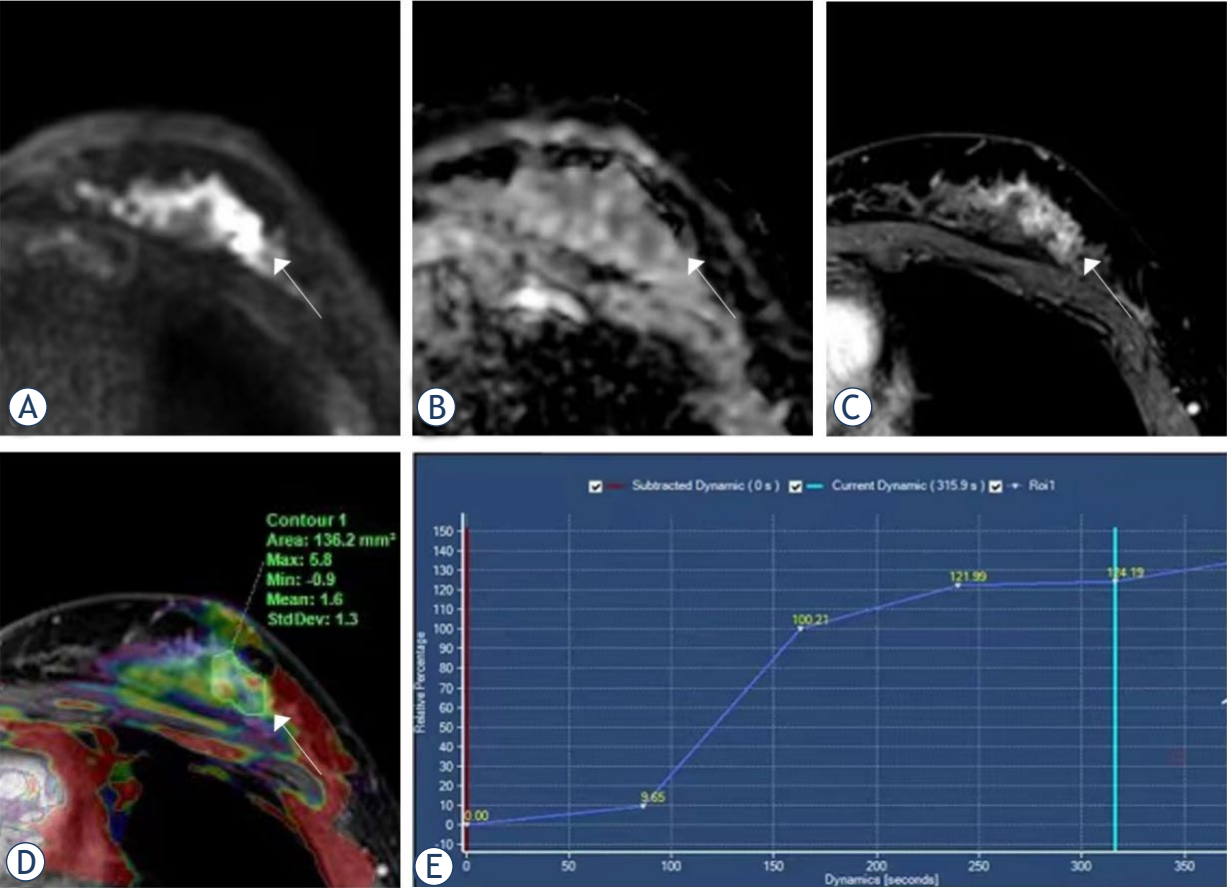
Magnetic resonance imaging (MRI) from a 26-year-old woman with intraductal papilloma. **(A)** Diffusion-weighted imaging (DWI); **(B)** apparent diffusion coefficient (ADC); **(C)** dynamic contrast-enhanced (DCE); **(D)** the amide proton transfer-weighted imaging (APTWI) pseudo-color image was merged with the DCE images and the APT value was 1.6%; **(E)** the type of time-intensity curves (TIC) was persistent.

Malignant group had significantly higher APT values than benign group (3.18 ± 1.07 and 2.01 ± 0.51, p < 0.001). It was found that the ADC values for the malignant group were lower than those for the benign group (1.13 ± 0.36 and 1.64 ± 0.41, p < 0.001). The type of TIC was considered statistically significant (p < 0.001, Table 4). Representative images for the malignant and benign groups are shown in Figures 2 and 3.

**TABLE 4.** Comparison of different parameters between benign lesions and malignant lesions

**Measurement index**	**Benign lesions (n = 14)**	**Malignant lesions (n = 28)**	**P**
**APT value**	2.01 ± 0.51	3.18 ± 1.07	< 0.001
**Type of TIC**			< 0.001
**I**	11(78.6%)	5(17.9%)	
**II**	3(21.4%)	20(71.4%)	
**III**	0	3(10.7%)	
**ADC value**	1.64 ± 0.41	1.13 ± 0.36	< 0.001

ADC = apparent diffusion coefficient; APT = amide proton transfer; Type of time-intensity curves (TIC): (I = Persistent; II = Plateau; III = Washout)

### ROC curve analysis

**FIGURE 4.**
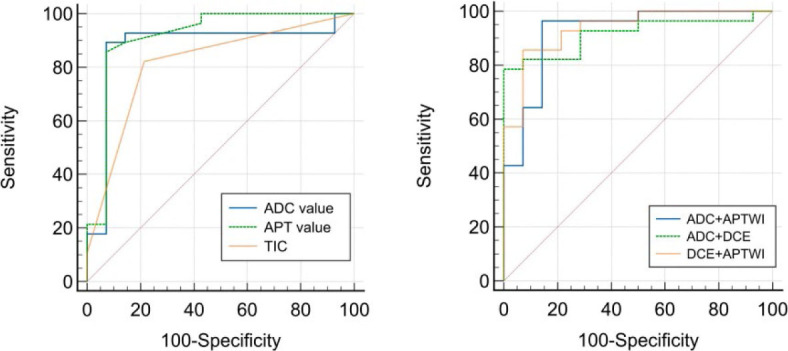
The graph displays ROC curves to evaluate the usefulness of various parameters for differentiating between malignant and benign lesions.

The APT value, TIC, and ADC value imaging shown AUC values of 0.915, 0.815, and 0.878, respectively, in distinction between the malignant lesions and the benign lesions. The AUC values of ADC + APTWI, ADC + DCE, and DCE + APTWI were 0.921, 0.916, and 0.936, respectively. However, only the differences between the AUC of TIC and DCE + APT and between the AUC of TIC and DCE + DWI were significant (Z = 1.987, p = 0.0470; Z = 2.049, p = 0.0405). The variations in AUC among various parameters are displayed in Table 5 and Figure 4.

**TABLE 5.** ROC analysis of the performance in separating breast cancer from benign lesions using various criteria and techniques alone or in combination

**Multi parameters**	**Cutoff**	**Sensitivity**	**Specificity**	**AUC**	**95%CI**
**Parameters**					
**APT value**	> 2.35	85.71%	92.86%	0.915	0.786–0.978
**TIC**	> 1	82.14%	78.57%	0.815	0.665–0.918
**ADC value**	≤ 1.26	89.29%	92.86%	0.878	0.739–0.958
**Methods**					
**ADC+APTWI**	/	85.71%	96.43%	0.921	0.795–0.982
**ADC+DCE**	/	71.43%	89.29%	0.916	0.788–0.979
**DCE+APTWI**	/	78.57%	92.86%	0.936	0.816–0.988

ADC = apparent diffusion coefficient; APTW = amide proton transfer-weighted imaging; AUC = area under the ROC curve; dynamic contrast-enhanced (DCE); TIC = time-intensity curves

### Correlation analysis

ER, PR, HER-2, and Ki-67 expression, as well as histological grade, did not significantly correlate with APT value (r = 0.254, 0.278, −0.222, −0.219, 0.029, respectively; p = 0.202, 0.161, 0.265, 0.273, 0.895, respectively).

## Discussion

In this prospective study, a pilot research of the viability of using APT value in conjunction with DWI and DCE sequences for the distinction between benign and malignant mammary lesions was successfully conducted. The correlation between the APT value and the pathological factors of breast cancer was explored. The feasibility of 3D APTWI MRI for the distinguishing between benign and malignant mammary lesions was demonstrated. With the clinical feasibility of APTWI in breast a concern among researchers, this study showed an excellent agreement in APT value measurement and the high diagnostic efficiency of APT values, similar to the efficiency of TIC, indicating that valuable diagnostic information could be obtained without using GBCA. Thus, APT value could be regarded as a non-invasive biomarker for differentiating mammary lesions.

In this study, the malignant lesions typically had higher APT values than the benign lesions, consistent with what is generally known about malignant tumors in other diseases.^22,23,24,25^ APTWI is a molecular MRI technique that is based on chemical exchange saturation transfer that could detect endogenous mobile proteins and peptides at low molecular concentrations. The high intensity in APTWI was made possible by the fact that malignant lesions were highly cellular and that several proteins were overexpressed in comparison to benign lesions. Due to the high levels of hemoglobin and albumin in blood, angiogenesis is another component that may contribute to enhanced protein signaling in malignancies.^13^ However, this discovery differs from the results of earlier research by Meng *et al*.^14,16^ They suggested that one reason could be that some benign lesions’ secretory abilities were intact, whereas the secretory abilities of some malignant lesions were damaged, leading to lower protein and polypeptide concentration. This difference could result from variations in study participants. Loi L *et al*. demonstrated that breast cancer displayed a substantially higher APT value than typical fibro-glandular tissue^26^, which fully confirms the results of the present study.

The findings showed that APT value has a strong diagnostic performance similar to TIC, and it could be employed for the differential diagnosis of mammary lesions. The combination of DWI and APT may increase the specificity of the diagnosis. DCE examination was conducted by injecting GBCA. As is well known, the injection of GBCA has many side effects, such as allergic as well as allergoid reactions, including anaphylactic reactions^27^, contrast-induced nephropathy^28^, nephrogenic systemic fibrosis^29^, and gadolinium retention/deposition.^30^ On the contrary, APTWI does not require the use of GBCA, thus saving costs and avoiding side effects. For patients with a history of allergies or those refusing to undergo DCE, APTWI could be a great substitute for DCE examination.

In this study, the APT value showed no correlations with ER, Ki-67 expression and histological grades, inconsistent with the results of previous studies. Liu Z *et al*. found a weakly positive connection between APT value and Ki-67 expression.^31^ A notable detail that they used a 20% threshold to report comparable APT values between groups with varying Ki-67 proliferation levels. This conflict could result from the differing Ki-67 proliferation index levels employed for group classification. Zhang N *et al*. reported that the APT value and ER expression had a negative connection, may be because ER inhibits lesion angiogenesis by regulating the production of the vascular endothelial growth factor.^32^ On the contrary, the results of syudy by Meng N *et al*. showed that there was no correlation between the APT value and ER expression.^14^ Consequently, a larger sample size is required for further research. Meng N *et al*. showed that only a weakly positive correlation existed between the pathogenic grade and APT value; they hypothesized that the cause is that high-level tumor cells have a high density, evident nuclear atypia, as well as increased tissue necrosis, all of which alters the proteins and peptides in the nearby microenvironment, in addition to the rate and distribution of internal water molecule diffusion and movement.^16^ Nevertheless, our grouping of histological grades was I, II, and III while their grouping of histological grades was low and high, which could explain the differences in the results. In accordance with previous researches, the APT value showed no correlations with PR and Her-2.^32,33^

This study has several limitations. First, breast lesions and some subtypes of breast cancer had relatively small sample sizes, which could cause the AUC of APTWI to be overestimated. Future research may also be warranted on the genomic subtyping capabilities of APTWI for breast cancers. Therefore, more research involving a bigger sample size is required. Second, the diagnostic efficacy of DCE assessed using only TIC curves may be underestimated. Liang X *et al*. recently revealed that the quantitative and semi-quantitative parameters of DCE provided great diagnostic performance. Third, breast tissue has a substantial amount of fat, which could affect the accuracy of APT values.^34^ Obviously, in the present study, patients with fatty breast tended to have poorer image quality. All CEST signals become essentially dependent on the amount of fat per voxel due to the ubiquitous fat signal's erroneous normalization of the Z-spectrum. Finally, bias may have existed in the selection of patients. Breast MRI is usually conducted in high-risk population.^35^ Patients receiving MRI in clinical practice had a considerably higher chance of malignant tumor than those receiving mammography or ultrasound, which could induce background bias (more malignant cases than benign cases).

## Conclusions

The capability of identifying benign from malignant breast lesions could be achieved using APTWI and DCE, and APTWI may be a great supplement or even replacement for DCE sequence. The findings still need to be confirmed by future investigations on patient cohorts with larger breast lesions.
